# Effect of surface grain boundary density on preosteoblast proliferation on titanium

**DOI:** 10.1080/21663831.2020.1744758

**Published:** 2020-04-07

**Authors:** Terry C. Lowe, Rebecca A. Reiss, Patrick E. Illescas, Casey F. Davis, Melanie C. Connick, Johnny A. Sena

**Affiliations:** a George S. Ansell Department of Metallurgical and Materials Engineering, Colorado School of Mines, Golden, CO, USA;; b Biology Department, New Mexico Institution of Mining and Technology, Socorro, NM, USA;; c National Center for Genome Resources, Santa Fe, NM, USA

**Keywords:** Cell proliferation, grain boundary density, ultrafine grain titanium

## Abstract

Studies since 2004 have shown that the cytocompatibility of ultrafine grain (UG) commercial purity (CP) titanium exceeds that of coarse grain (CG) CP titanium (Ti) by 30% to 20-fold. To isolate the factors affecting this large reported variability of CP titanium’s cytocompatibility, discs of UG and CG titanium were fabricated with controlled texture and roughness. The discs were seeded with MC3T3-E1 pre-osteoblastic cells and cultured for 72 h. The proliferation of cells on polished UG-Ti exceeded unpolished CG-Ti 3.04-fold. Cell proliferation was found to correlate with a new biophysical parameter, the average grain boundary length per surface-attached cell.

## IMPACT STATEMENT

By eliminating the influences of texture and roughness we have isolated the positive correlation between MC3TE-E1 cell proliferation on titanium and grain boundary length per surface-attached cell.

## Graphical ABSTRACT

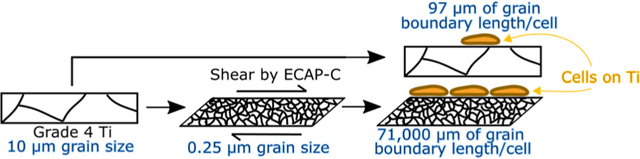

## Introduction

Titanium (Ti) alloys are widely used for dental implants [1] and orthopaedic devices [2]. Thus, the design of microstructures and surfaces of Ti medical implants has been the focus of research to enhance the integration of Ti with bone tissue [3]. Surface treatments can create desirable nanoscale structures on surface on Ti, as reviewed by Wang, *et al.* [4] and Kunčická, *et al.* [3], but enhancing titanium’s strength for load-bearing applications also requires fine-scale volumetric features rather than surface-only modifications. The strength [5], corrosion resistance [6], and cytocompatibility [7,8] of commercial purity (CP) Ti can be enhanced by introducing sub-micron and nanometer-scale microstructural features via Severe Plastic Deformation (SPD) methods to produce ultrafine grain (UG) titanium. Continuous Equal Channel Angular Pressing (C-ECAP) [9], a specific form of SPD, can fabricate long, high-strength Ti rods suitable for making medical devices [10]. C-ECAP reduces grain size, alters grain boundary structure, and increases the areal surface density of grain boundaries [11].

The surface of Ti is of special interest since it forms the interface to the physiological environment surrounding medical prosthetics. Studies have demonstrated that the growth of preosteoblasts and other cell types on UG-Ti can be increased from 1.3-fold (30% increase) up to 20-fold (1900%) compared to conventional coarse grain (CG) Ti [7,8]. This enhancement of cell viability on SPD-processed metals has been attributed to the effects of surface energy [12], topography [13], grain size [14], and crystallographic texture [15]. Medvedev et al. [16] measured the effects of all of these variables on the viability of cells grown on CG-Ti and UG- Ti. They found that the viability of SaOS-2 cells and adipose-derived mesenchymal stem cells (adMSC) was greatest for substrates polished to nanoscale smoothness (average mean roughness *R*_*a*_ *<* 4 nm) for both cell types and they recommended further investigation to quantify non-topological factors on such smooth surfaces. Baek et al. [17] systematically varied the crystallographic texture, grain size, surface energy, and roughness of Grade 2 Ti. They found greater proliferation of MC3T3-E1 cells on UG-Ti surfaces, which had higher surface energy, roughness, and more numerous grain boundaries. However, because they varied surface energy and roughness together, they were not able to differentiate the contributions of surface energy from surface roughness.

Here we follow up on the work of Medvedev, et al. and Baek, et al. by designing our experiments to isolate grain size-related effects on the extent of MC3T3-E1 preosteoblast attachment and proliferation on CG-Ti and UG-Ti. We fabricated substrates with nearly identical crystallographic textures and nanoscale smoothness so that we can focus our attention solely on the influence of grain size and the density of grain boundaries on cell response. Our goal is to test the hypothesis that grain boundaries influence cell proliferation, independent of texture and surface roughness.

## Materials and methods

Rods of mill-annealed 15 mm diameter commercially pure Grade 4 titanium (Carpenter Technology Corporation, Heat H10803) were cut to lengths between 150 and 450 mm for C-ECAP processing. Table 1 lists the alloy composition. The rods were pre-heated to temperatures between 150°C and 200°C and subject to C-ECAP for 4 sequential passes through a 120° die with 90° rotations between each pass to achieve a total shear strain of4.6. After C-ECAP, all rods were drawn and centerless-ground to a diameter of 13.3 mm.

The rods were sectioned transversely into thirty-two 2 mm thick discs and ground using a progression of SiC abrasive papers (grit 120, 240, 400, 800, 1200, 2500, 4000). Half of the discs were additionally polished to nanometric smoothness using a Buehler Vibromet™ 2 vibratory polisher with a suspension of 20 nm colloidal silica for 12 h to 24 h. Discs were cleaned using organic soap, sonicated for 10 min in high purity isopropanol, rinsed, and surface topographies were documented using a Digital Research Nanoscope IIIa atomic force microscope (AFM).

The discs were sterilized by 12-hour exposure to 100% isopropanol. Mouse preosteoblasts from the cell line MC3T3-E1 sub-clone 4 (American Type Culture Collection (ATCC), Rockville, MD) were cultured at 37°C at 5% CO_2_ in Alpha Minimum Essential Medium (MEM *α*, GIBCO, Grand Island, NY) supplemented with 10% fetal bovine serum (FBS, GIBCO, Grand Island, NY) and 1% penicillin/streptomycin (Cellgro, Manassas, VA). Discs were seeded in 24-well cell culture plates (USA Scientific, CC672–7524) at a density of 1.5 × 10^4^ cells per well and incubated for 24 and 72 h. Culture media, along with unattached cells, were removed and fresh media was added at 24 h intervals.

Cell DNA was stained with DAPI (4’,6-diamidino-2-phenylindole) at a concentration of 10 μg/ml and intra-cellular lipids were stained by Nile Red (9-diethylamino-5H-benzo[alpha]phenoxazine-5-one) at a concentration of 10 μg/ml. Plates were then incubated in darkness at 37°C for 15 min. Cells were counted using a haemocy-tometer. Each disc was imaged with an Eclipse LV100 fluorescence microscope equipped with a Hamamatsu ORCA-RF camera and Nikon Plan Fluor 10x objective lens using 478–495 nm wavelength filters for DAPI and 532–587 nm wavelength filters for Nile Red. Triplicate colorimetric images were rendered using ImageJ software to quantitate the stained cells [18].

Cells were treated with 2% glutaraldehyde for 15 min and then dehydrated with ethanol rinses. After the final rinse, the cells were dried on the discs overnight. The discs were sputter-coated with platinum and imaged with a Hitachi S-2460N scanning electron microscope (SEM). For three replicates of each Ti substrate condition a JEOL JSM-7000F SEM with an EDAX Hikari Pro Electron Backscatter Diffraction (EBSD) Camera G was used to measure grain size distribution, grain boundary lengths and misorientations, and crystallographic texture. EBSD measurements were repeated at multiple electron microscopy magnifications, between 300x and 35,000x to confirm quantitative microstructural analysis statistics and ensure the absence of scan-size effects. Energy Dispersive Spectroscopy (EDS) was also conducted to measure the possible effects of segregation of elements present in the alloy.

## Results

**Figure 1.**
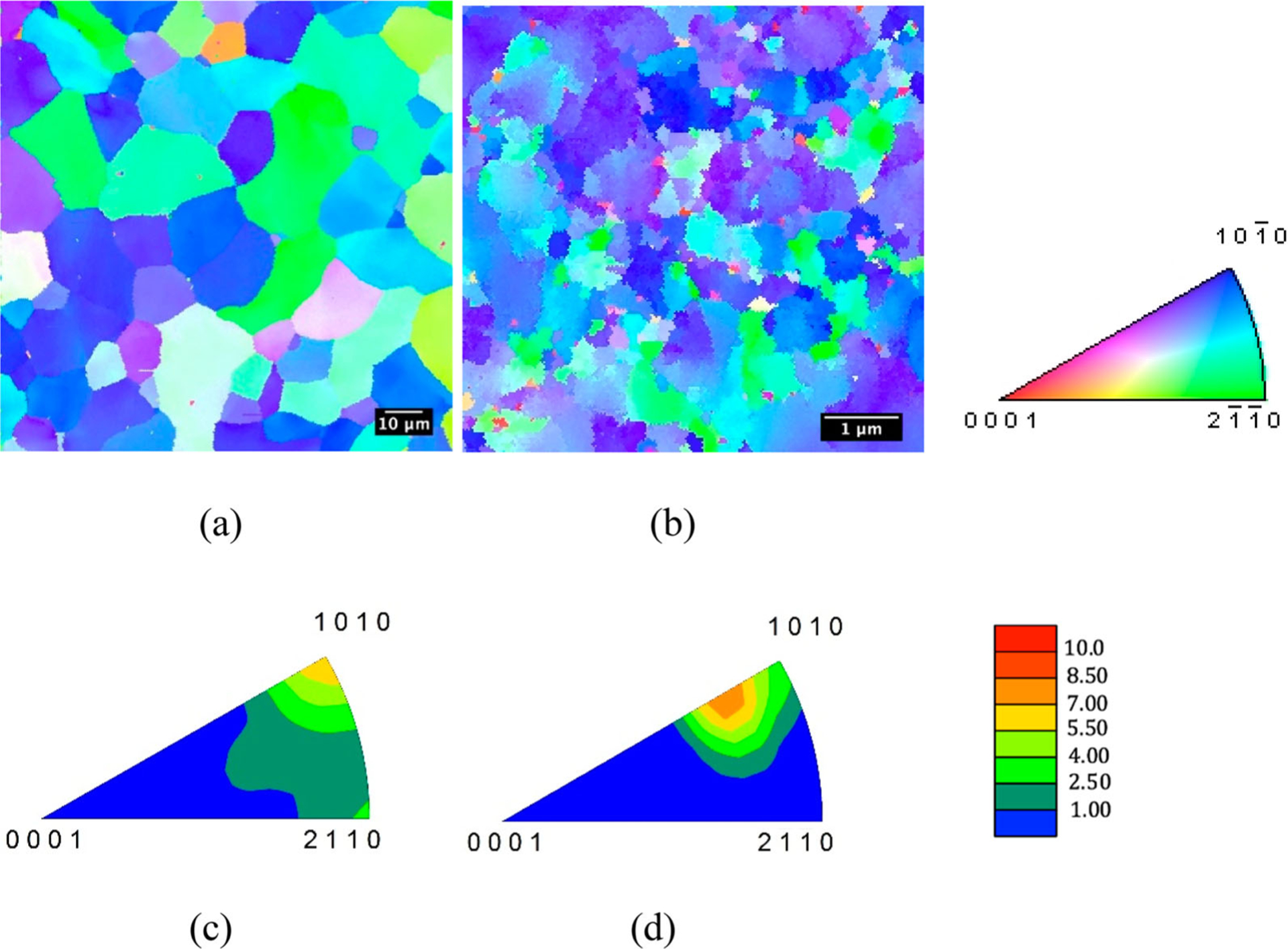
Rod/extrusion axis inverse pole figure maps of (a) CG-Ti (1000x magnification) and (b) UG-Ti (20,000x magnification); and corresponding rod/extrusion axis inverse pole figures for (c) CG-Ti and (d) UG-Ti.

Grain orientation maps measured via EBSD show the structures and crystallographic textures for CG-Ti and UG-Ti in Figure 1. Deformation-induced refinement of the grain size is apparent in the inverse pole figure map in Figure 1b, though some micron-sized grains remain in the UG-Ti, even after the large strains imposed through C-ECAP.

**Figure 2.**
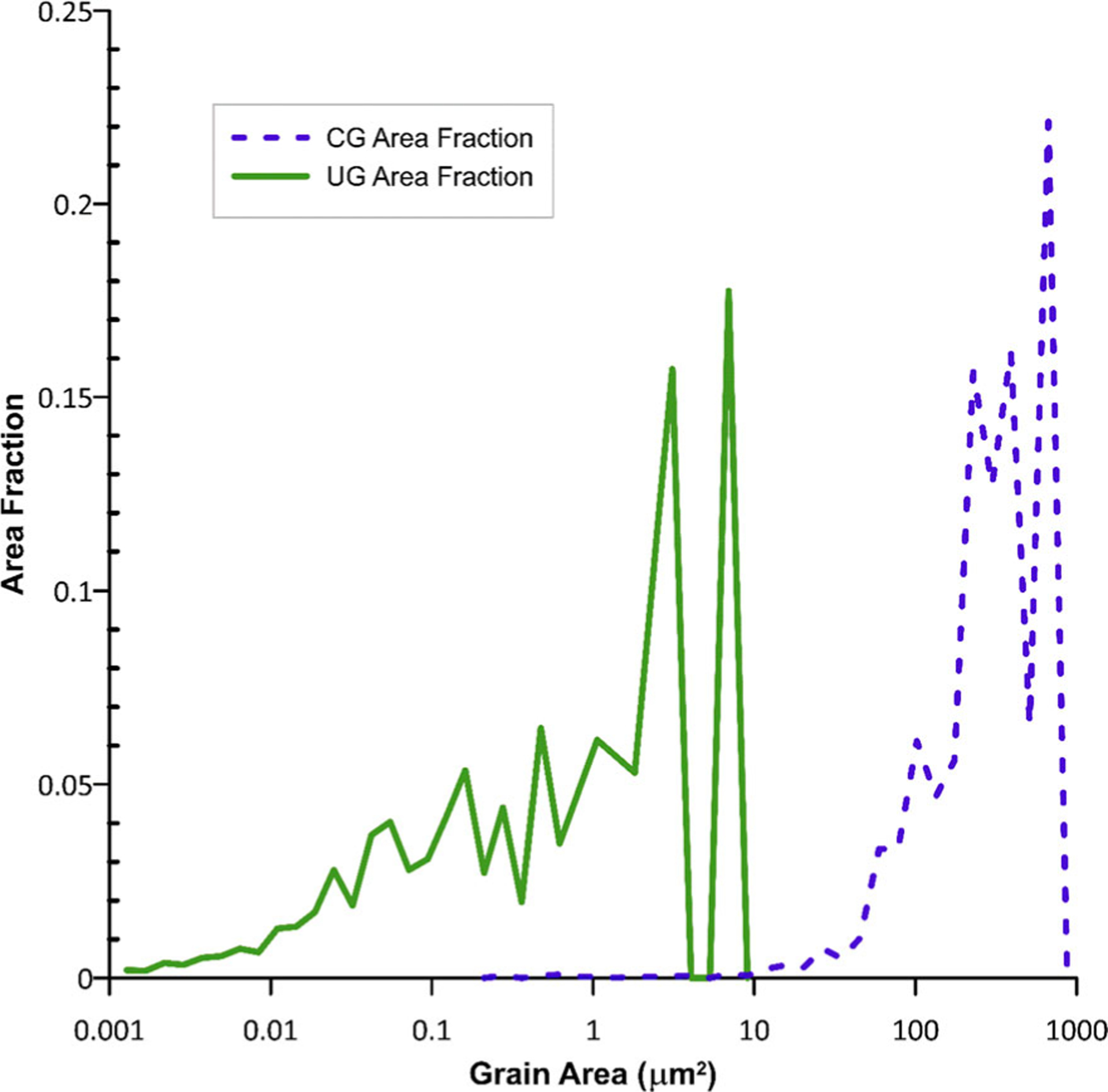
Grain area distributions for CG-Ti and UG-Ti.

The average grain size was 10.9 μm +/− 7.3 μm for CG-Ti samples compared to 0.24 μm +/−0.38 μm for UG-Ti samples, a 45-fold difference. The difference in grain size distributions for CG-Ti and UG-Ti surfaces is shown in Figure 2. The average grain area for the CG substrates was 125.6 μm^2^ compared to 0.0752 μm^2^ for UG substrates. The inverse pole figures in Figures 1c and 1d show the preference for $\left\{10\bar{1}0\right\}$ prism plane normals align with the rod axis for both CG and UG samples. The similarity in textures was achieved by additional rod drawing reductions as the final deformation processing step after the C-ECAP. The maximum times-random texture intensity was 6.7x for the CG-Ti samples, and slightly higher, 8.3x for UG-Ti samples. For the UG-Ti, the orientation having the maximum intensity was shifted on average approximately 14.6° from the $\left[10\bar{1}0\right]$ direction toward the [0001] direction.

Atomic Force Microscopy measurements showed the average arithmetic mean roughness R_a_ for the assectioned unpolished discs was 40.2 nm +/− 24 and 70.2 nm +/− 36 nm for the CG-Ti and UG-Ti, respectively. In contrast, the average R_a_ for the polished discs was 0.24 nm +/− *<* 0.2 and 0.30 nm +/− *<* 0.2 nm for the CG and UG discs, respectively. The polished discs were nanometrically smooth. For the unpolished discs, the actual surface areas were only marginally greater than the projected areas, 3.02% higher for the CG-Ti samples and 2.59% higher for the UG-Ti samples. For the polished discs the differences in actual and projected surface areas were minuscule, 0.019%. Thus, the effective area available for cells to contact surfaces was not significantly different for the unpolished substrates compared to the polished substrates.

**Figure 3.**
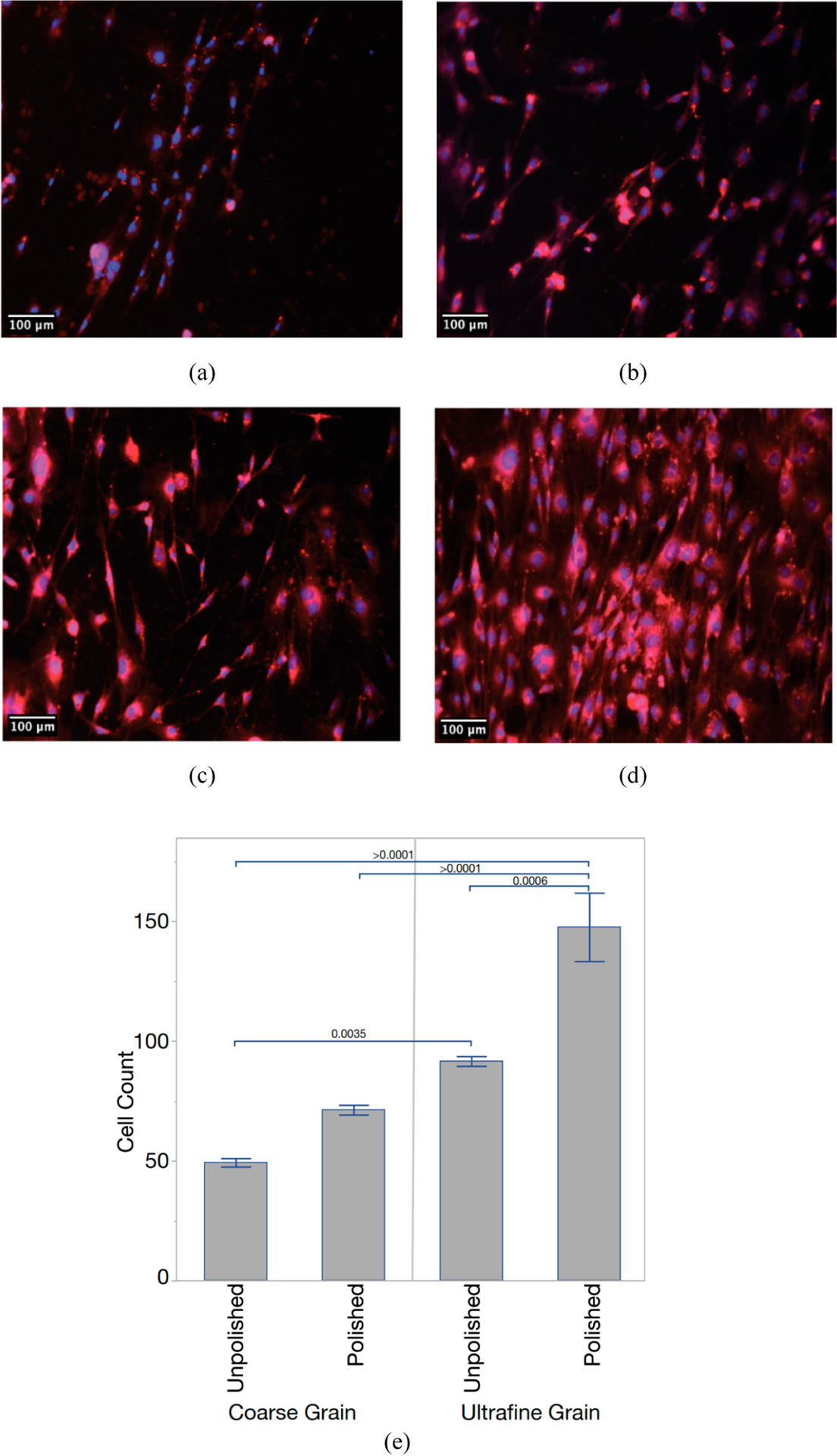
Fluorescent images of mouse preosteoblasts after 72 h for (a) CG-Ti unpolished, (b) CG-Ti polished, (c) UG-Ti unpolished, and(d) UG-Ti polished. Mean cell counts (e) for all four states, with *p*-values for significant pairwise comparisons shown.

DAPI- and Nile Red-stained preosteoblasts, as shown in Figure 3, were counted on all samples after 24 (not shown) and 72 h. After 24 h, an average of 20 cells was observed on the four surfaces and there were no statistically significant differences.

After 72 h, an average of 72 cells was observed on polished CG-Ti versus 149 on polished UG-Ti, and 49 on unpolished CG-Ti versus 91 on unpolished UG-Ti. The mean number of surface-attached MT3T3-E1 cells was 1.9-fold and 2.1-fold higher compared to CG-Ti after 72 h of cultivation on unpolished and polished substrates, respectively. The differences observed after 72 h are due to increased proliferation, not initial adhesion to the substrate. The combined effects of UG-Ti and polishing increased the average number of attached cells3.04-fold.

## Discussion

The Ti grain structures imparted by C-ECAP are comparable to those found by others in UG-Ti [19–21]. Like- wise, the enhanced cell attachment and proliferation are consistent with prior cell proliferation studies [7,8]. However, in the present study, the crystallographic textures and surface topographies for the CG and UG states were intentionally made similar. The maximum intensity of the $\left\{10\bar{1}0\right\}$ texture was 8.3 times-random for the UG samples comparable to the 6.7 times-random measured for the CG samples. Since the $\left\{10\bar{1}0\right\}$ texture fiber is rotated towards the [0001] direction in the UG sample, the intensities of the $\left\{10\bar{1}0\right\}$ texture fibers for CG and UG are both between 4 and 6 times-random around the *<* $10\bar{1}0$ fiber direction. Thus, prospective effects of differences in crystallographic texture on surface energy were minimized. Likewise, the effects of surface topography were eliminated for the nanometrically smooth substrates.

The areal densities of grain boundaries intersecting the surfaces differed for the CG and UG samples. From the EBSD analysis, an average total grain boundary length per unit surface area of 0.19 μm/μm^2^ was measured on CG-Ti compared to 12.62 μm/μm^2^ measured on UG-Ti. Thus, the average areal density of grain boundaries is 67.5 times higher on the UG-Ti surface compared to the CG-Ti surface. This difference is the greatest contrast between CG-Ti and UG-Ti in this study.

The average distance between grain boundaries with respect to the average dimensions of attached cells differs greatly for CG-Ti and UG-Ti. Recall that the average grain sizes were 10.9 μm and 0.24 μm for CG-Ti and UG-Ti, respectively. The typical widths of attached preosteoblast cells, as seen in the images in Figure 3 were between 6 μm and 20 μm. Pseudopodia of these cells extended to lengths up to 250 μm. Thus, for the CG-Ti, a typical attached cell covers 1–2 grains by their width, and up to approximately 20 grains by their length. In contrast, for a UG-Ti surface the same typical cell would cover between 20 and 70 grains by their width and up to approximately 900 grains by their length. Thus, UG-Ti presents a far larger number of grain boundaries to surface-attached cells. The grain boundaries on surfaces that were made nanometrically smooth by polishing are readily assessable to proteins and surface-attached cells since they are not obscured by larger-scale topographical features. To explain the trend in cell proliferation in Figure 3 we introduce a parameter that combines a physical characteristic of the substrate, the average Grain Boundary Length (GBL) per unit area, with a cytological characteristic, the average area on the substrate covered by surface-attached cells. The ratio of these quantities yields the average grain boundary length per average-sized cell, which we designate GBL/Cell. Values of GBL/Cell are given in Table 2 for all four combinations of titanium surface roughness and grain size.

As hypothesized, the greatest lengths of grain boundaries are found under cells attached to UG-Ti. Polished surfaces exhibit higher cell counts than unpolished surfaces, but this difference is not as great as the effect of grain boundary length. The average preosteoblast cell size varies by 1 order of magnitude from the CG unpolished condition to the UG polished condition. However, the ratio of total average grain boundary length per cell varies by almost 3 orders of magnitude. A linear least squares correlation between the cell count and log_10_(Grain Boundary Length/Cell) fits well, with a correlation coefficient R^2^ of 0.88. Cell proliferation increases monotonically with increasing grain boundary density underneath the cells. This trend is present regardless of surface roughness, though polished surfaces consistently displayed higher cell counts and larger cell sizes. We expect that the roughness of the unpolished sample surfaces reduces the access of cells to grain boundaries because of geometric effects of the topography. Analysis of SEM micrographs of cells on the substrates revealed that cells occupy concave valleys in the unpolished surfaces, a location preferable to attachment on the convex peaks of surface striations created by machining of the samples.

**Figure 4.**
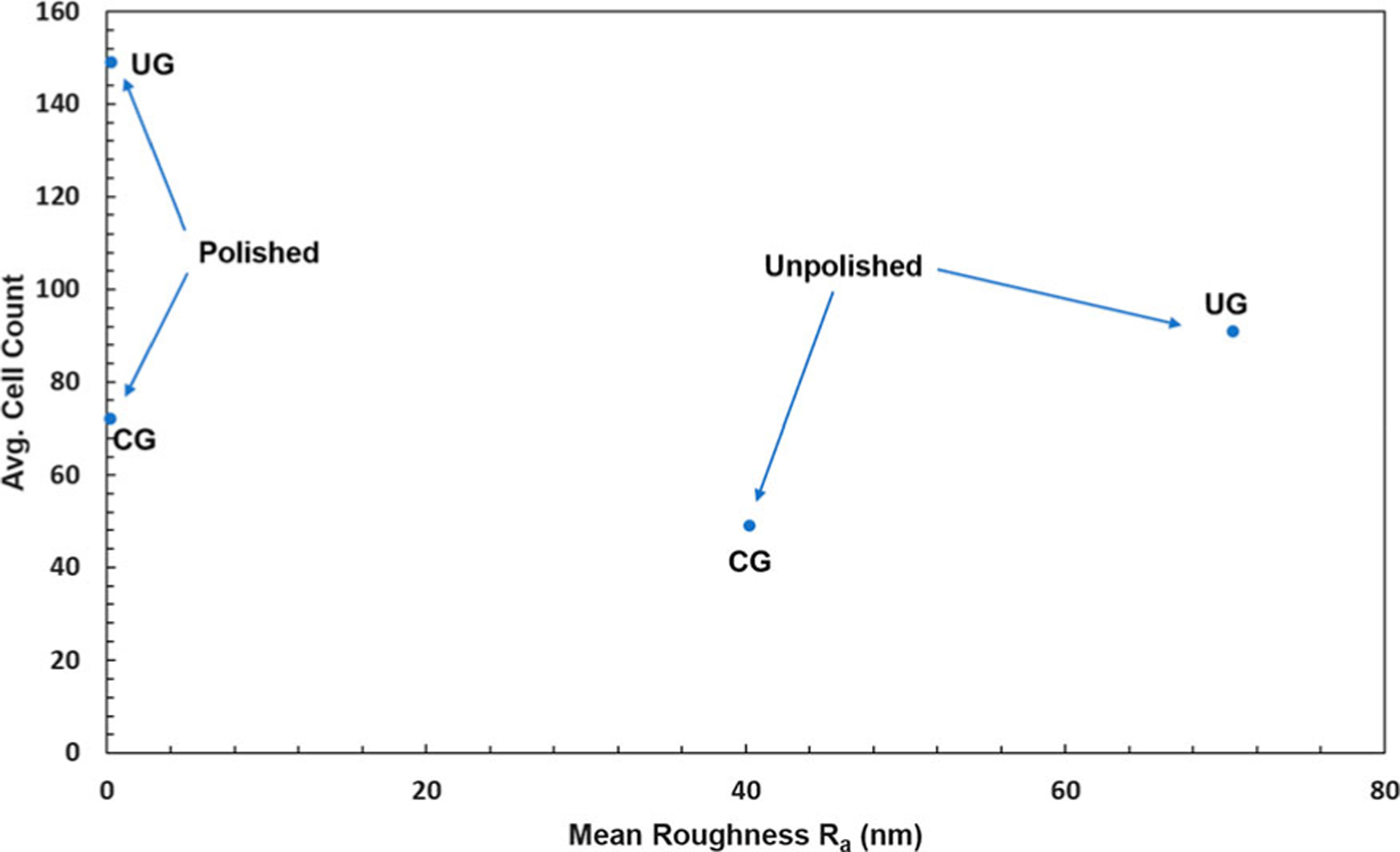
Relationship between the average number of attached MC3T3-E1 cells and average arithmetic mean roughness R_a_.

Figure 4 displays the variation of average cell count with surface roughness measured by AFM. While the polished samples had essentially identical roughness, with *R*_*a*_ *<* 1 nm, significantly higher cell counts were found on the UG-Ti substrates compared to the CG-Ti substrates. Similarly, the unpolished UG-Ti samples also had significantly higher cell counts, though the R_a_ values were significantly different, 40.2 nm +/− 24 nm for CG- Ti versus 70.2 nm +/− 36 nm for UG-Ti. These results are congruent with Medvedev, et al. for Grade 4 Ti [16] and Baek et al. for Grade 2 Ti [17]. However, in the experiments reported here the effects of surface roughness are absent in the polished samples and cannot contribute to the difference we found in the cell counts for UG and CG samples.

**Figure 5.**
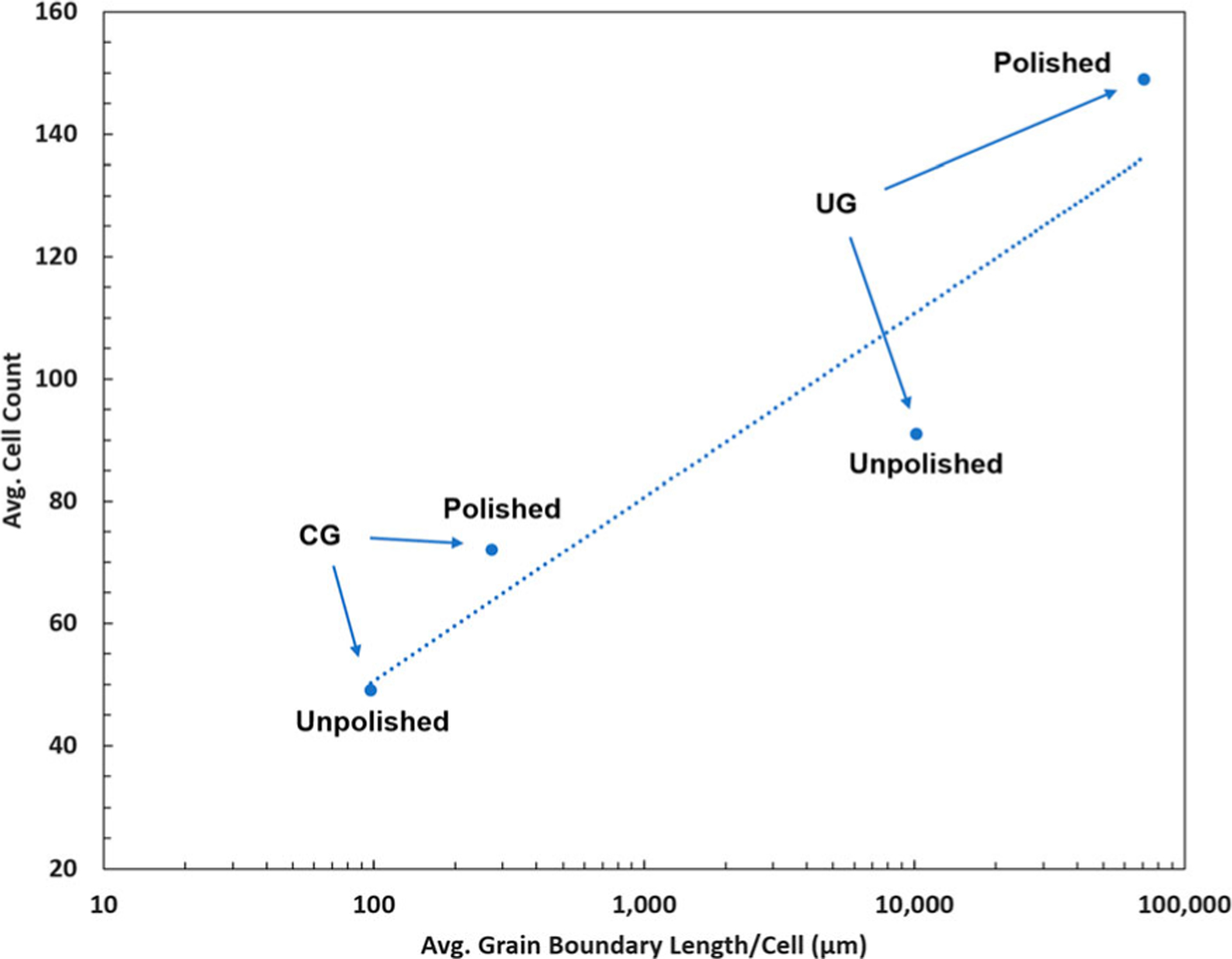
Attached number of MC3T3-E1 cells after 72 h increases with GBL/Cell, the average length of grain boundaries per attached cell on the substrate surface.

The observed correlation between grain boundary length and cell proliferation shown in Figure 5 reshapes our outlook on hypotheses for the underlying causes and mechanisms. One explanation for this correlation is the prospective influence of the higher energy of grain boundary regions compared to the intra-grain matrix. These energy differences have the potential to alter the work function and distribution of charge within the surface oxide, which may influence the adsorption of proteins or interaction with transmembrane adhesion proteins such as integrins. This possibility has been explored by Ercan, et al. [22]. Also, it is notable that65.6% of the grain boundaries on UG-Ti had intercept lengths less than 82 nm. This population of closely spaced grain boundary regions on the UG-Ti samples over-laps with the expected average separation distance of cell focal adhesions and their integrin ligands. Le Saux et al. reported this separation to be 44 nm for endothelial cells [23]. This connection supports a hypothesis that grain boundaries have a greater opportunity to influence mechanotransduction and signaling on smooth UG-Ti. This mechanism is also consistent with results from a companion study of the sequencing of the RNA from the cells grown on nanometrically smooth UG- Ti and CG-Ti surfaces. These experiments implicate an interaction of integrins with grain boundaries as the factor that increases cell proliferation through established mechanosensation and transduction pathways (to be reported in a separate manuscript in preparation). Whether this is due to physical characteristics of grain boundaries that increase protein adsorption or direct interaction of the cell with the boundaries remains to be determined.

Another alternative mechanism by which grain boun daries may influence cell proliferation is the possibility of microsegregation of alloying elements or impurities. Locally high concentrations of these elements could present chemical differences in grain boundary regions. However, in our study Energy Dispersive Spectroscopy (EDS) measurements at 20,000× to 35,000× magnifications did not show evidence of microsegregation of Fe, O, N, or C on any substrate (data not shown).

Following the work of Ercan et al [22], it is plausible that the underlying mechanisms and effects of varying surface roughness differ for surfaces with roughness R_a_ in the 1–10 nm range compared to the 10–100 nm range. However, Figure 5 shows that the number of cells relative to the grain boundary length correlates with cell proliferation for the entire range of surface roughness in the present study. For the polished samples with *R*_*a*_ *<* 1 nm, the grain boundaries presented no significant topographical features to proteins or cells on the surface, indicating that the grain boundaries themselves have a role in cell attachment and proliferation. Additional experiments are needed with more levels of surface roughness and grain boundary densities to verify this conclusion. However, the present experiments are the first to isolate the positive correlation of grain boundary length with cell attachment and proliferation through the parameter GBL/Cell which represents the average total grain boundary length per surface-attached cell.

## Conclusion

Preosteoblasts from the MT3T3-E1 line were cultured for 72 h on four states of CP Ti substrates: CG and UG, and with or without polishing to nanometric smoothness. The mean numbers of surface-attached MT3T3-E1 cells on UG-Ti were 1.9-fold and 2.1-fold higher compared to CG-Ti after 72 h of cultivation on unpolished and polished substrates, respectively. The combination of having ultrafine grains and nanosmooth surfaces produced the greatest increase in the number of surface-attached cells, by a factor of 3.04 compared to unpolished CG-Ti. A new biophysical parameter, the average grain boundary length per surface-attached cell (GBL/Cell), was proposed to explain the results.

Grain boundary length per cell was computed by combining data from image analysis of surface-attached cells with the EBSD data on grain boundary length. The average grain boundary length per unit surface area was 13.8 times greater for UG-Ti compared to CG-Ti. The average number of attached MT3T3-E1 cells increased monotonically with GBL/Cell. A linear correlation was found between the average number of surface-attached cells and the logarithm of GBL/Cell (linear least square correlation coefficient *R*^2^ = 0.88), showing that cell attachment and proliferation correlate with the density of grain boundaries on the surface of Ti.

## Figures and Tables

**Table 1.** Composition of Grade 4 Titanium.

Element	C	Fe	0	N	Y	H	Other Elements	Total of Other Elements	Ti
Wt.%	0.051	0.12	0.36	0.010	< 0.0004	0.0023	<0.1	<0.30	Bal

**Table 2.** Measured surface-attached cell and grain boundary parameters.

	Mean # of Attached Cells	Avg. Substrate Area Covered Per Cell (μm2)	Avg. Grain Boundary Length per Unit Area (μm/μm2)	GBL/Cell, Avg. Grain Boundary Length per Cell (μm/cell)
CG-Ti, Unpolished	49	518.2	0.187	97.1
CG-Ti, Polished	72	1457.5	0.187	273.0
UG-Ti, Unpolished	91	807.3	12.62	10,189.3
UG-Ti, Polished	149	5615.7	12.62	70,881.5
